# Reverse-Type Takotsubo Cardiomyopathy: A Case Report

**DOI:** 10.7759/cureus.104992

**Published:** 2026-03-10

**Authors:** Jeffrey A Tyre, Samuel A Hampton, Shaniece Bond, Kirsten Grubb

**Affiliations:** 1 Emergency Department, Magnolia Regional Health Center, Corinth, USA; 2 Emergency Medicine, Ross University School of Medicine, Jackson, USA; 3 Emergency Medicine, Arkansas College of Osteopathic Medicine, Paragould, USA

**Keywords:** : acute coronary syndrome, apical hypokinesis, left ventriculogram, reverse takotsubo, takotsubo cardiomyopathy (ttc)

## Abstract

Reverse Takotsubo cardiomyopathy (TCM), also known as inverted TCM, is a rare variant of TCM. Typical TCM affects the apex of the heart, while reverse takotsubo affects the base of the left ventricle. Reverse TCM is characterized by basal hypokinesis and apical hyperkinesis. It typically occurs in younger individuals with a higher prevalence in men who have experienced emotional or physical stress. The case presented below is very atypical, considering it occurred in a 74-year-old female. TCM can also be associated with intracranial hemorrhage, anesthesia, or other neurological conditions. Treatment usually entails supportive management, focusing on heart failure therapy and addressing the underlying trigger. Conservative management with beta-blockers, angiotensin-converting enzyme (ACE) inhibitors, and angiotensin receptor blockers can be used to mitigate catecholamine surges and sympathetic overstimulation. Common complications of reverse TCM include myocarditis, effusions, and left ventricular (LV) thrombi. While both forms can be severe, reverse TCM is associated with less hemodynamic compromise and faster recovery, typically marked by a return to normal left ventricular function within a few weeks.

A 74-year-old woman with bilateral lower extremity cellulitis and no history of coronary artery disease presented via EMS to the emergency department (ED). The patient complained of chest pain following a verbal and physical altercation. The electrocardiogram en route showed ST elevation, and the patient was administered four sublingual nitroglycerin tablets and 324 mg of aspirin. In the ED, the patient's ECG showed a normal sinus rhythm with right axis deviation, and troponin was elevated. Computed tomography angiography of the chest was negative for pulmonary embolism. The patient was admitted to the medicine service with a cardiology consult. Cardiology suspected TCM, which was confirmed by a left ventriculogram.

We discuss the typical presentation of the atypical diagnosis of TCM. Of the atypical forms of the disease, the most common is reverse TCM. The clinical manifestation often mimics acute coronary syndrome with chest pain, elevated cardiac biomarkers, and ECG changes, but coronary angiography usually reveals no significant obstructive findings. This case report highlights the importance of maintaining a broad differential diagnosis in patients presenting with chest pain and of recognizing those at risk for TCM.

## Introduction

Takotsubo cardiomyopathy (TCM), also known as broken heart syndrome, Takotsubo syndrome, and stress cardiomyopathy, was first described in Japan in 1990. Named after the angiography findings resembling a Japanese octopus trap, it is described as transient regional left ventricle dysfunction in the absence of coronary artery disease in the affected segment [1]. As of January 2022, Takotsubo accounts for 1%-2% of suspected acute coronary syndrome cases that were troponin positive, with increasing incidence attributable to clinical awareness of the condition and modern stressors. TCM is found in 6% of women who undergo emergent angiography due to a suspected STEMI. In Western countries, TCM is more common in post menopausal women, while it is more common in Japanese men compared to Japanese women [1,2]. TCM pathologic *framework *is theorized to be caused by catecholamines and ischemic changes, but there is no one pathophysiologic explanation to explain TCM in its entirety [3]. In a study of 1750 patients, 75.9% presented with chest pain, 46.9% experienced dyspnea, 7.7% percent experienced syncope, with other studies reporting heart failure symptoms such as orthopnea and pulmonary edema [2,4]. Among patients, 27.7% presented following an emotional trigger, 36% following a physical stressor, 7.8% experienced both emotional and physical stressors before symptom onset, and 28.5% presented without any identifiable stressor [4]. Multiple variants have been discovered, one of which is reverse TCM, characterized by basal wall ballooning that resolves spontaneously. The proportion of patients with reverse TCM out of all TCM variants is less than a quarter of all cases. The recurrence rate is around 10%. 

Troponin I, troponin T, and creatine kinase are elevated to levels comparable with those seen in acute myocardial infarction (MI), peaking within the first 24 hours. Brain natriuretic peptide (BNP) and N-terminal pro-B-type peptide peak at 48 hours after the onset of symptoms, reflecting left ventricular impairment. Imaging helps differentiate TCM from an acute MI, myocarditis, and other similar appearing presentations. Coronary angiography can reveal akinesis or dyskinesia of the mid- and apical left ventricle segments, accompanied by hyperdynamic basal segments. Other imaging modalities include ECG, transesophageal echocardiography (TEE), cardiac CT angiography (CTA), and cardiac magnetic resonance imaging (MRI). The Takotsubo International Registry has developed tools to aid in identifying TCM on clinical presentation: a bedside scoring system, the InterTAK diagnostic score, which does not require imaging, and the InterTAK Diagnostic Criteria [2].

Treatment for TCM aims at managing and preventing complications, including heart failure, cardiogenic shock (5%-10%), thromboembolism (2%-9%), left ventricular outflow tract obstruction, and intramyocardial hemorrhage or rupture [5]. There is no standardized long-term treatment for TCM; however, studies suggest benefits from prescribing beta-blockers in recurrent TCM and persistent anxiety, improved one-year survival with the use of ACE inhibitors (ACEi) and angiotensin II receptor blockers (ARBs), and evaluation for psychiatric comorbidities and substance abuse [5,6].

This case report aims to highlight the clinical presentation of an atypical variant of TCM, thereby improving recognition, avoiding unnecessary invasive procedures and testing, preventing complications, and enhancing patient outcomes. Many patients recover normal cardiac function with close cardiology monitoring.

## Case presentation

A 74-year-old female presented to the Emergency Department (ED) via emergency medical services (EMS) for chest pain. The patient had a verbal and physical altercation that resulted in her being pushed to the ground before developing chest pain. The chest pain was described as a burning sensation and mainly affected the left side of her chest.

EMS arrived at the scene and placed the patient on the stretcher. The patient had difficulty with ambulation after her recent fall from the altercation, and she had been suffering from chronic right lower leg cellulitis. En route to the ED, the patient received four nitroglycerin sublingual tablets and 324 mg of chewable aspirin. EMS also obtained a rhythm strip that showed ST elevations concerning for ST-elevation myocardial infarction in the lateral leads. Unfortunately, the rhythm strip was not uploaded to the chart, so a copy cannot be provided.

Further history obtained in the ED revealed that the patient was not taking any blood thinners and had no prior history of heart disease. Physical examination showed an elderly female who was mildly anxious but not in acute distress. Cardiac auscultation revealed a normal heart rate, rhythm, and heart sounds. Pulmonary auscultation demonstrated clear breath sounds bilaterally. The patient’s right leg, from the knee to the foot, showed diffuse swelling, weeping, and sinus tracts. In the Emergency Department, blood pressure remained normal, with an initial reading of 116/71 mmHg.

**Figure 1:**
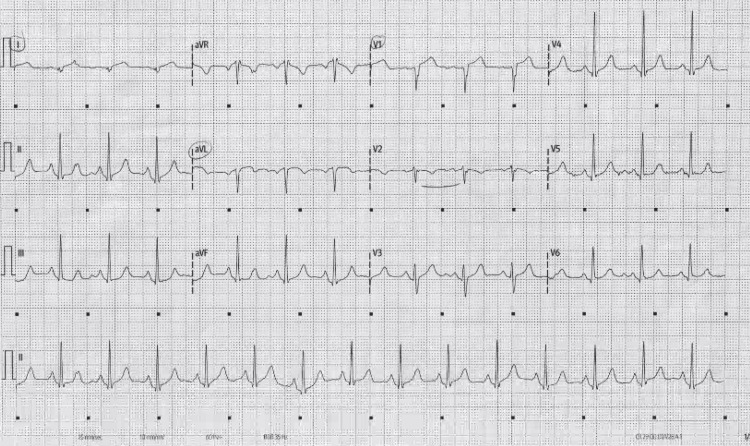
Initial ECG on arrival. The ECG demonstrates T-wave inversion in V2 and minimal ST-segment elevation in leads I and V1.

Electrocardiogram (ECG) in the emergency department revealed sinus rhythm with a ventricular rate of 87 bpm and right axis deviation (Figure 1). The PR interval, QRS duration, and QTc interval were all within normal limits. Minimal ST-segment elevation was noted in leads I and V1, with T-wave inversion in V2. A repeat ECG demonstrated ST-segment elevation in leads I and V2. Complete blood count revealed mild anemia but was otherwise unremarkable. Troponin was elevated at 3.20 ng/mL (normal <0.012 ng/mL), with a repeat value of 3.17 ng/mL four hours after the initial measurement. Chest CTA was ordered due to concern for pulmonary embolism given the patient’s history of right lower extremity pain. The study was negative, showing no evidence of aortic dissection or pulmonary embolism. The patient’s HEART score was 6.

Cardiology was consulted from the ED due to concern for acute coronary syndrome (NSTEMI) and recommended that the patient be admitted to the medicine service with cardiology consultation. Cardiology suggested that the patient was likely experiencing Takotsubo cardiomyopathy and that emergent heart catheterization was not indicated at this time. The medicine team was contacted, and the patient was admitted to the floor.

Cardiology performed a transthoracic echocardiogram three hours after initial presentation. Results revealed an ejection fraction of 35%-40% and hypercontractility of the apex with hypokinesis of the basal left ventricular wall (Video 1). 

**Video 1:** Transthoracic echocardiogram showing apical hypercontractility and basal hypokinesis of the left ventricular wall.

The cardiologist later performed a left heart catheterization 14 hours after initial presentation. A left ventriculogram revealed reverse Takotsubo cardiomyopathy, with a hypercontractile apex and akinetic base (Video 2). No significant coronary artery disease or coronary obstruction was noted.

**Video 2:** Left ventriculogram: Visualizing a hypercontractile apex and hypokinetic base of the left ventricle.

Besides echocardiogram and left-heart catheterization, the patient’s hospital stay was uneventful. She was diagnosed with vitamin B12 deficiency. The patient remained stable. Repeat ECG normalized, with no ST-segment elevations, T-wave inversions, or Q waves noted. The patient’s blood pressure remained normal to low, so an ACEi was not initiated. She was encouraged to follow up as an outpatient but was subsequently lost to follow-up. At discharge, the patient was started on atorvastatin and vitamin B12 supplementation.

## Discussion

In this case, a 74-year-old female developed acute chest pain following a physical and emotional stressor, namely, a physical altercation and subsequent fall. Her initial prehospital ECG showed ST-segment elevations suggestive of an STEMI, prompting administration of aspirin and nitroglycerin. However, subsequent ECG in the ED did not show persistent ST abnormalities, and cardiac biomarkers were notably elevated (troponin 3.2 ng/mL), raising suspicion for myocardial injury without a clear ischemic etiology.

Of particular clinical interest is the interplay of emotional stress and chronic physical illness (right lower extremity cellulitis with associated swelling and impaired ambulation) as potential contributing factors. Emotional triggers such as interpersonal conflict are well-established precipitants of TCM, especially in elderly females - a population that comprises the majority of TCM cases. The patient’s demographic profile (elderly, postmenopausal female) and context (sudden emotional and physical trauma) are consistent with reported risk factors for TCM.

Transthoracic echocardiography revealed reduced ejection fraction (35%-40%) and regional wall motion abnormalities in the apical and lateral walls. The diagnosis was confirmed by left heart catheterization, which demonstrated a reverse Takotsubo pattern, characterized by basal hypokinesis and a hypercontractile apex, in the absence of significant coronary artery disease. Reverse TCM, although less common than the typical apical variant, is more frequently reported in younger patients and those with neurological events, although it can still occur in older populations. Typical TCM features apical hypokinesis, whereas the reverse type shows basal hypokinesis.

Notably, this patient’s HEART score was 6, placing her in the high-risk category for a major adverse cardiac event (MACE), and given her presentation, she required inpatient monitoring. While TCM is generally considered a reversible condition with a favorable prognosis, complications such as heart failure, arrhythmias, and left ventricular thrombus may occur, particularly in patients with reduced ejection fraction.

This case underscores the importance of maintaining a broad differential in patients presenting with chest pain, especially when classic ischemic findings are not fully supported by subsequent diagnostic imaging. In patients with psychosocial stressors and no prior cardiac history, TCM should remain a key diagnostic consideration. Early cardiology consultation, echocardiography, and coronary angiography were crucial in arriving at the correct diagnosis and preventing unnecessary intervention for presumed ACS. Diagnosis of Takotsubo is important for further monitoring of cardiac function.

## Conclusions

This case highlights the importance of considering TCM in elderly patients presenting with acute chest pain following significant emotional or physical stress, especially when initial ECG and biomarker findings suggest acute coronary syndrome but are not corroborated by angiographic evidence of obstructive coronary disease. Reverse TCM, though less common, should be recognized as a distinct variant with its own characteristic imaging findings. Reverse TCM should be considered in a high-risk patient (elevated heart score) who has developed chest pain after a significant life stressor. Prompt identification and differentiation from true myocardial infarction are essential to avoid unnecessary interventions and to guide appropriate management. Awareness of this condition can facilitate timely diagnosis and supportive care, leading to favorable outcomes in most patients. Diagnosis of this condition allows outpatient cardiology follow-up for continued monitoring and serial echocardiograms.
